# Coupling Socioeconomic and Lake Systems for Sustainability: A Conceptual Analysis Using Lake St. Clair Region as a Case Study

**DOI:** 10.1007/s13280-013-0432-4

**Published:** 2013-08-15

**Authors:** Georgia Mavrommati, Melissa M. Baustian, Erin A. Dreelin

**Affiliations:** Center for Water Sciences, Michigan State University, 301 Manly Miles Building, 1405 S. Harrison Rd, East Lansing, MI 48823 USA

**Keywords:** Coupled human and natural systems, Lake St. Clair, Clinton River watershed, Ecosystem services, Stakeholders, Sustainability

## Abstract

Applying sustainability at an operational level requires understanding the linkages between socioeconomic and natural systems. We identified linkages in a case study of the Lake St. Clair (LSC) region, part of the Laurentian Great Lakes system. Our research phases included: (1) investigating and revising existing coupled human and natural systems frameworks to develop a framework for this case study; (2) testing and refining the framework by hosting a 1-day stakeholder workshop and (3) creating a causal loop diagram (CLD) to illustrate the relationships among the systems’ key components. With stakeholder assistance, we identified four interrelated pathways that include water use and discharge, land use, tourism and shipping that impact the ecological condition of LSC. The interrelationships between the pathways of water use and tourism are further illustrated by a CLD with several feedback loops. We suggest that this holistic approach can be applied to other case studies and inspire the development of dynamic models capable of informing decision making for sustainability.

**Electronic supplementary material:**

The online version of this article (doi:10.1007/s13280-013-0432-4) contains supplementary material, which is available to authorized users.

## Introduction

The concept of sustainability includes various assumptions about the relationship between human-made and natural capital as well as the needs and preferences of current and future generations. Divergence in these assumptions has led to two opposing schools of thought: strong and weak sustainability (Neumayer 2010). The main difference between the two paradigms is that strong sustainability rejects the major assumption of weak sustainability in that natural capital can be substituted by human-made capital (Daly 1996). With regard to the preferences of future generations, strong sustainability assumes preferences are unknown and unknowable, whereas weak sustainability anticipates that they will be similar to those of the present generation (Bithas 2008). Another fundamental difference between the two schools of thought is the way the benefits of future generations are taken into account. Low and zero discount rates are proposed by strong sustainability approaches, while positive discount rates are used by weak sustainability approaches, thereby reflecting different ethical considerations (Sumaila 2004; Howarth 2009). Implicitly, weak sustainability views human and natural systems as independent, while strong sustainability views them as interdependent systems (Mavrommati and Richardson 2012).

Recent evidence suggests that further deterioration of natural systems may provoke tremendous impacts to humanity (Rockstrom et al. 2009; Folke et al. 2011), thus the sustainability of human and natural systems cannot be examined separately. Under the current model of economic development and increasing human population size, the dependence of socioeconomic activities on natural resources is becoming so high that we cannot ignore the ‘limits to growth’ stemming from natural systems (Burger et al. 2012). Therefore, applying sustainability at an operational level requires deep understanding of the structure, processes, and interactions of human and natural systems, the so-called coupled human and natural systems (CHANS) approach (Liu et al. 2007).

A CHANS approach can enhance our understanding regarding the dependence of socioeconomic systems on natural systems and enable decision makers to design more effective policies for managing ecosystem services (ES) (Pickett et al. 2005; Liu et al. 2007; Alberti et al. 2011). CHANS frameworks constitute the basis for building integrated models that specify factors and processes for applying the principles of sustainability (Pickett et al. 2005; Carpenter et al. 2009). Conceptual frameworks are essential starting points to illustrate components, pathways, and hypothesized responses among the subsystems in a CHANS framework (Alberti et al. 2011). Delineating the couplings taking place among human and natural systems is a complicated process that requires both extensive scientific and applied knowledge from various disciplines (Ostrom 2009). Ostrom (2009) and Ostrom and Cox (2010) suggest a general framework for studying sustainability of socio-ecological systems and identifying variables that might affect resource users to “avert the tragedy of the commons” by designing and implementing costly governance systems. Interacting with diverse stakeholders is necessary to incorporate expert knowledge, especially at various stages of the research, planning, and building of frameworks (Maxwell 1996; Schmolke et al. 2010; Cumming 2011). The use of conceptual frameworks also assists and encourages discussion across scientific disciplines and stakeholder sectors and is therefore a critical prerequisite for building complex models (Heemskerk et al. 2003; Alberti et al. 2011).

The most widely known CHANS framework is the Millennium Ecosystem Assessment (MEA) framework (2003) that includes four subsystems: ES, direct and indirect drivers of change, and human well-being (HWB). The innovative element of the MEA framework is the linkage between HWB and ES. Based on this framework, a revised CHANS framework has been proposed by emphasizing the fundamental role that changes in HWB have in the formulation of environmental policies (Stevenson 2011). The revised MEA framework highlights the importance of environmental policy for regulating the impacts of human activities on ES and sustaining HWB. Our goal was to build a framework that identifies the key parameters and pathways affecting the function of both natural and human systems and incorporates the knowledge of stakeholders. We began by further refining the MEA and revised MEA frameworks (Stevenson 2011) by explicitly including ecosystem condition responses to the stressors produced by human activities and how those responses may affect ES. We applied our conceptual framework to a real-world, dynamic case study in order to provide an example of the necessary steps for developing a CHANS approach. This approach is based on the premise that the maintenance of minimally-disturbed ecological condition of western Lake St. Clair (LSC) is necessary for ensuring the satisfaction of human needs now and in the future (Holden and Linnerud 2007). This is the first time a framework that couples the socioeconomic and natural systems has been developed for the LSC region (Clinton River watershed and western shore of LSC). Previous work in this watershed reported findings on the conditions of human or natural systems without providing insights for the couplings and feedbacks between them (Bricker et al. 1976; Selegean et al. 2001; Francis and Haas 2006; van Hees et al. 2010).

Developing a framework as a tool to understand the interactions between humans and the environment is needed in this area for several reasons. First, even with serious threats (i.e., beach closures) to the ES in the region, there are still local initiatives that promote outdoor recreational activities (i.e., Macomb County Blue Economy Initiative, LSC Tourism Initiative) and encourage their increased use. With respect to sustainability, there is a need to understand how ES that support and are affected by recreational activities can be maintained in the long-term. Second, the Clinton River, which flows into and influences the western shore of LSC, is one of the most ecologically impaired rivers in the state of Michigan based on fish and macroinvertebrate communities (Riseng et al. 2010) implying the need for better understanding of pollutant sources and impacts. Third, LSC is surrounded by counties with high human population densities that have an increased demand on the ES provided by the LSC region and to a great extent is linked historically with the socioeconomic evolution of the city of Detroit (SEMCOG 2002; Baustian et al. unpublished).This multidisciplinary approach is not intended for only this case study, but can be applied in other systems to assist scientists and stakeholders in building dynamic models that incorporate interactions between systems and ES and apply the principles of sustainability at an operational level.

## Methods

### Case Study

**Fig. 1:**
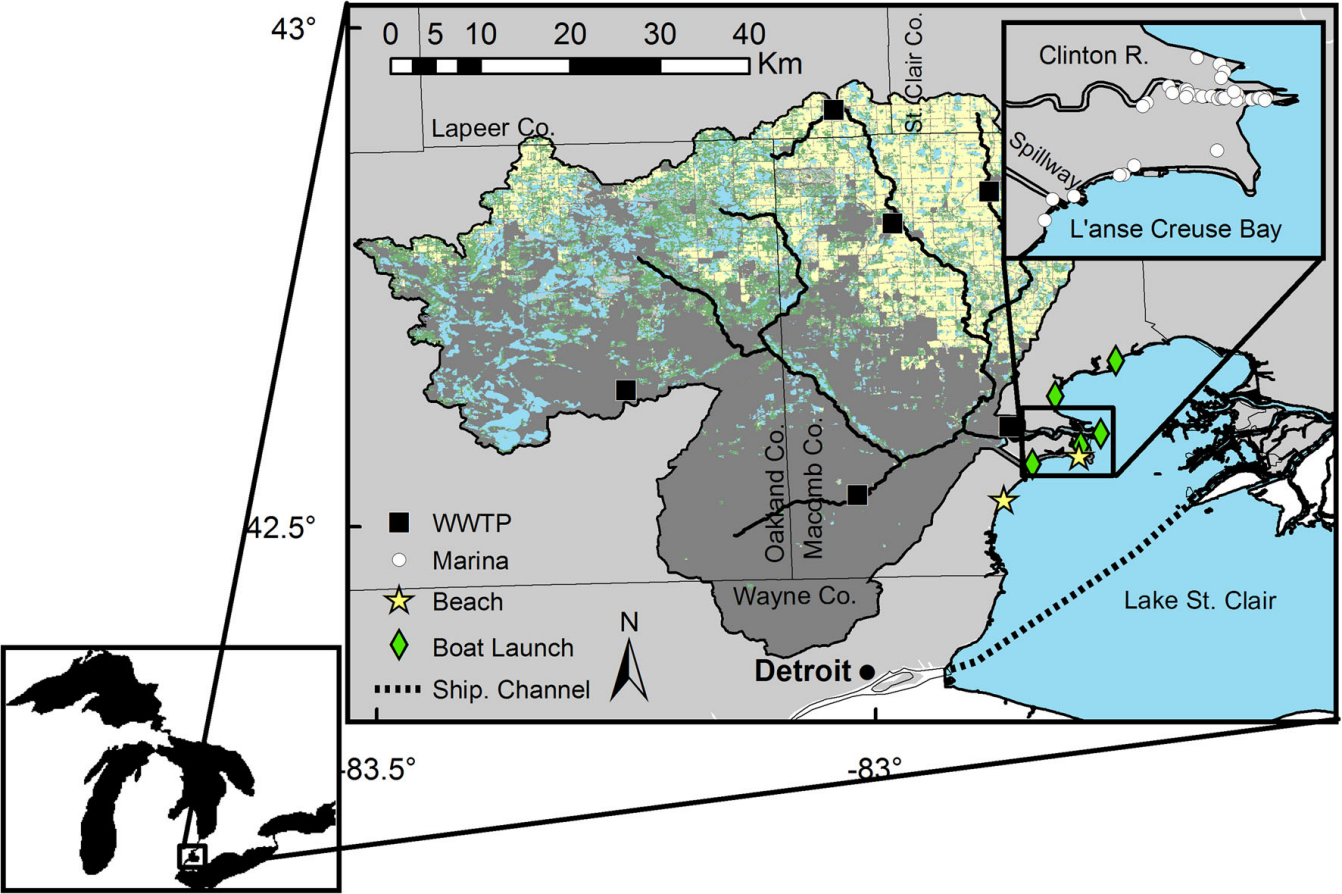
Map of the land use (developed *dark gray*, open water and wetlands *blue*, forest *green*, and agriculture *yellow*) of Clinton River watershed based on the 2006 NLCD (Fry et al. 2011) and the western shore of LSC, a connecting channel in the Laurentian Great Lakes system. *Upper map* is a close-up of the mouth of the Clinton River. *WWTP* wastewater treatment plants. Marinas, beaches (nearest to river), and boat launches indicate the recreational use of the area

LSC is considered to be the “heart of the Great Lakes” because it provides an important connecting channel in the Laurentian Great Lakes system (Fig. 1). It links Lake Huron to Lake Erie via the St. Clair River to the north and Detroit River to the south; these waters are known as the Huron-Erie corridor. The prevailing winds and the St. Clair River water that enters the lake from the north produce a coastal current that tends to direct the Clinton River plume to the south (Schwab et al. 1989; Anderson and Schwab 2011) along the coastline, including the sandy beaches. LSC provides essential ES to the region, such as drinking water and recreational activities. These ES are also popular, as demonstrated by the total number of visitors at Metro Beach (Lake St. Clair Metropark Beach) which is estimated >200 000 people per month during the summer months.

The Clinton River is approximately 128 km long and is located in eastern Michigan, USA (Fig. 1). It drains an area of about 1980 km^2^ that includes the northern suburbs of the Detroit Metropolitan area and the majority of Oakland and Macomb Counties and smaller areas of St. Clair, Lapeer, and Wayne Counties (Jweda and Baskaran 2011). The Clinton River watershed encompasses approximately 60 cities, towns, and villages and is home to more than 1.5 million people (van Hees et al. 2010) who are mainly employed in automotive manufacturing and associated services. As of 2001, land use within the watershed was approximately 31 % forest, 26 % developed, 23 % agricultural land, and 14 % grasslands/open areas (Healy et al. 2008). The counties within the Clinton River watershed have one of highest densities of permitted point-source pollution facilities (>75 National Pollutant Discharge Elimination System permits per county) in the Great Lakes Basin (US Government Accountability Office 2005) and according to the last census the growth rate of population and number of households is positive (2.6 and 8.7 %). The river and spillway, which was constructed in 1952 to relieve flooding, empty into the western shore of LSC near L’anse Creuse Bay.

Six wastewater treatment plants (WWTPs) with a total carrying capacity 264 675 m^3^ day^−1^ operate in the watershed and serve more than 500 000 people. The rest of the population is connected to the Detroit’s’ WWTP or has septic systems. An important issue in the region is the replacement of the aging wastewater infrastructure as inflow, infiltration and combined sewer overflows (CSOs) impact water quality and human health (SEMCOG 2001). Financial constraints pose a major challenge on developing funding schemes for replacing wastewater infrastructure and sustain water quality in the area.

The Clinton River watershed and the western lake shoreline, downstream of the river mouth have been designated as an Area of Concern since 1987 under the Great Lakes Water Quality Agreement (International Joint Commission 2006). There are currently 41 Areas of Concern listed in the Great Lakes region due to one or more of 14 beneficial use impairments (BUIs) (http://www.epa.gov/glnpo/aoc/clintriv.html). Examples of the BUIs in the Clinton River watershed and western shore of LSC (8 of 14 BUIs) are: eutrophication or undesirable algae, degradation of fish and wildlife populations, and beach closures.

### Research Phases

#### Phase I: Developing a Revised Conceptual Framework and Applying It to a Case Study

The construction of the conceptual framework was based on the principles and assumptions underlying the MEA and revised frameworks (Millennium Ecosystem Assessment 2005; Stevenson 2011) as well as existing theory and research concerning the coupling of human and natural systems that considers the possible linkages, interdependencies, and feedbacks between the natural and socioeconomic systems (Liu et al. 2007; Walsh and McGinnis 2008; Alberti et al. 2011). A conceptual framework was developed reflecting our understanding of how the socioeconomic and lake system interact with respect to the maintenance of the ecological condition for ES and HWB and then modified based on the published literature for the Clinton River and western shore of LSC (Bricker et al. 1976; Healy et al. 2008; van Hees et al. 2010).

#### Phase II: Testing and Refining the Framework by Hosting a 1-Day Stakeholder Workshop

**Fig. 2:**
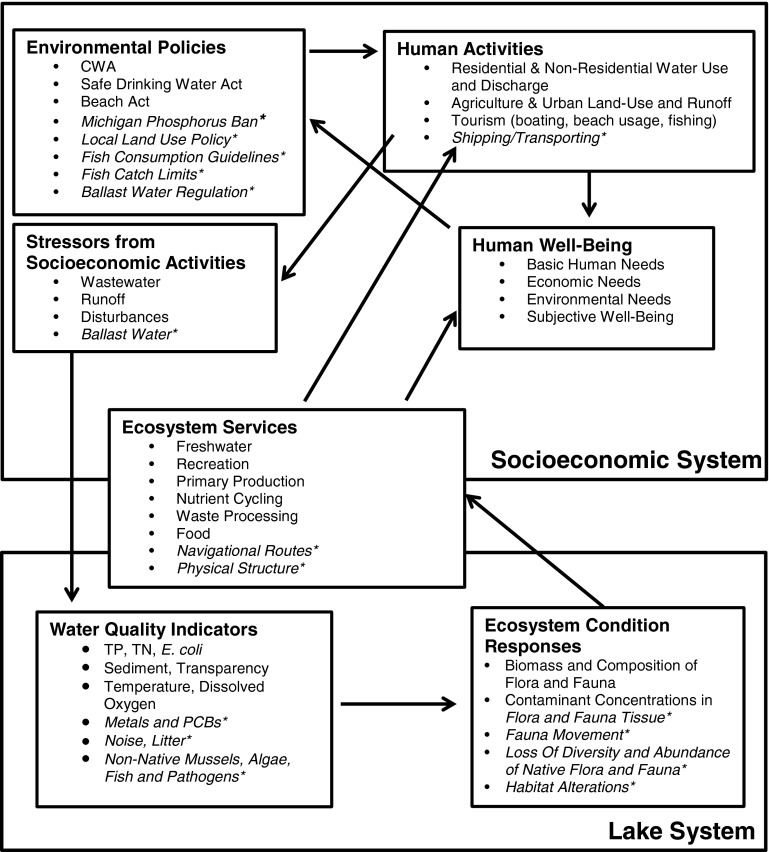
A CHANS framework for the Clinton River watershed and western shore and LSC. * indicates stakeholders’ input. *TP* total phosphorus, *TN* total nitrogen

A 1-day workshop was held to elicit the expert knowledge of professionals working in the LSC region. Fifteen stakeholders from a pool of 30 individuals with various expertise (e.g., ecology, community planning, engineering, economics, public health) and organizations (e.g., public utilities, universities, county, state and federal agencies), brought many years of experience in working in the area to discuss CHANS. Invited stakeholders were not aware of our hypothesized parameters and linkages in the conceptual framework because we did not want to influence their opinions. Before the workshop, we met frequently with a professional facilitator to help plan, organize and think about expected outcomes. We also emailed the stakeholders a short assignment beforehand that consisted of filling out their own conceptual diagram (the boxes of the two systems with no parameters or arrows) (see Appendix S1). We asked them to think about the indicators of ecosystem health, the socioeconomic activities that impact them and how ecological condition affects HWB. The main objective of workshop was to test and refine the parameters and linkages in the conceptual framework. The workshop goals were achieved by three exercises in which the stakeholders worked in small groups. First, each group listed key parameters of concern in the socioeconomic and lake (i.e., natural) systems (Fig. 2). Second, as a team they added arrows to show how the parameters affected each other (Fig. 2). Lastly, a discussion followed about potential future management options. Based on this stakeholder input, we refined the conceptual framework by compiling the parameters and arrows from the groups into one diagram. We compared and contrasted our framework to the results of the workshop and focused on parameters and linkages identified as important for LSC region by both the stakeholders (see Fig. 2 with *) and our team. Most of our stakeholders commented that they were not familiar or accustomed to working with CHANS conceptual frameworks and they expressed that the workshop gave them the opportunity to think about CHANS and discuss more broadly about their own discipline and how it connects to others.

#### Phase III: Creating a Causal Loop Diagram Based on System Dynamics

A causal loop diagram (CLD) was developed based on system dynamics methodology to better understand the complexity and feedback loops among some of the parameters indicated in the conceptual framework. We used Stella Software (Version 10.02) to create the CLDs. One of most useful features provided by the software is the sensitivity analysis that occurs after running the model to learn if basic patterns of results are sensitive to changes in the uncertain parameters (Ford 1999).

## Results

### Phase I and Phase II: A CHANS Framework for Lake St. Clair

The proposed framework has two main systems: the socioeconomic and the lake (Fig. 2). The socioeconomic system is composed of four interrelated sub-systems: human activities, stressors from socioeconomic activities, HWB, and environmental policies (Fig. 2). The lake system is described through two subsystems: water quality indicators and the ecosystem condition responses.

ES link both systems (Fig. 2). The lake system provides ES that directly benefit the socioeconomic system in two ways: first, by providing inputs to economic activities (e.g., commercial water use) and second, by influencing HWB (e.g., provisioning drinking water to meet basic biological human needs). Human activities taking place in the socioeconomic system indirectly affect ES by the production of stressors that decrease water quality and disturb the ecosystem condition, which in turn affects the provision of ES.

Using the CHANS framework and its application to LSC, we identified four main pathways and seven essential categories that describe key relationships between the socioeconomic and lake systems (Table 1). All of these pathways describe how human activities degrade the ecological condition and ES provided by the lake, but the pathways also indicate how humans depend on these natural systems for their well-being. Human activities might affect different ES than the one they depend on and for this reason two columns of ES are presented in Table 1.

**Table 1:** Four pathways that connect the socioeconomic system, ecosystem services and lake system in the Clinton River watershed and western shore of LSC

Socioeconomic system (SS)				Ecosystem services (ES)		Lake system (LS)	
Human activities (HA) that produce stressors	Stressors from HA	Human well being components from HA and ES	Environmental policies regulating stressors	ES as inputs to SS	ES impacted by LS responses	Water quality indicators that are impacted by stressors	Ecosystem condition response to water quality indicators
Pathway 1: residential and non-residential, water use and discharge	Wastewater: BOD, TSS, nutrients, waterborne pathogens, temperature, metals, PCBs	Basic human needs, economic needs, environmental needs, subjective well-being	Clean Water Act, Safe Drinking Water Act, MI Phosphorus Ban	Freshwater (D), waste processing (ID), nutrient cycling (ID)	Freshwater, primary production, nutrient cycling, waste processing, recreation, biodiversity, food	TP, TN, *E. coli*, sediment, transparency, temperature, dissolved oxygen, mercury, PCBs	Biomass and composition of flora and fauna; concentrations in flora and fauna tissue
Pathway 2: land use (agr and urban runoff)	Runoff: nutrients, TSS, waterborne pathogens, altered hydrology	Basic human needs, economic needs, subjective well-being	Clean Water Act, local land use policy	Freshwater (D), recreation, aesthetics, navigational routes, physical structure	Same as Pathway 1	TP, TN, *E. coli,* sediment, transparency, temperature, dissolved oxygen, metals, PCBs	Same as Pathway 1
Pathway 3: tourism (boating, beach usage and fishing)	Disturbances: physical, noise, sediment, submerg. veg., litter; discharges from vessels	Economic needs, subjective well-being	Beach Act, Fish catch limits, fish consumption guidelines	Recreation (D), primary production (ID), nutrient cycling (ID), waste processing (ID), food (D), aesthetics, navigational routes (D)	Freshwater, recreation, primary production, biodiversity, food	Noise, transparency, litter, concentrations of anti-fouling paints	Flora and fauna movement and distribution, habitat alterations, flora and fauna tissue; fish communities composition and abundance
Pathway 4: shipping/transporting	Ballast water: non-native mussels, algae, fish, and pathogens; dredging	Economic needs, environmental needs, subjective well-being	Ballast Water Regulation, Clean Water Act	Physical structure (D), navigational routes (D)	Recreation, food, biodiversity	Non-native organisms; sediment	Diversity and abundance of native flora and fauna; habitat alterations

*D* direct use of ES, *ID* indirect usages, *BOD* biological oxygen demand, *TSS* total suspended solids, *TP* total phosphorus and *TN* total nitrogen

### Description of the Pathways

The first pathway is the human use of water and, consequently, the production of pollutant loads through residential sewage and non-residential discharge (Table 1). In this respect, water is an input to the socioeconomic system which is transformed into a pollutant load (wastewater) output to the Clinton River.

The second pathway is non-point source pollution related to land use, which is a significant component of contaminant loads in the runoff from the Clinton River watershed (Table 1). Approximately one-fourth of the Clinton River watershed is agricultural (Fig. 1). The type of products (e.g., fertilizers and pesticides) and cultural practices used in agricultural watersheds drives pollutant loads (Jung et al. 2008). Another one-fourth of land use in the watershed is developed and thus stormwater runoff from impervious surfaces is also a key stressor and source of pollution to the river and lake (Environmental Consulting and Technology 2007). It is important to note that mixtures of pollutants derived from various sources can interact in the environment and have the potential to produce adverse effects to ecological and human health (Ravichandran 2004; Sumpter et al. 2006; Barber et al. 2013).

Tourism is the third pathway where humans and the environment significantly interact (Table 1). The importance of this activity to HWB was reiterated by the stakeholders. Beach usage, boating and fishing can pollute and disturb the natural environment by causing wave action, visual disturbances, noise pollution, resuspension of sediment and submerged vegetation, and increase litter (fishing line, tackle, food wrappers, etc.) into the river and lake (Mosisch and Arthington 1998; Graham and Cooke 2008) (Table 1). Conversely, tourism is an important part of the economy of LSC region and it is estimated that beach closures in LSC results in a welfare loss $13.89 per person per trip (Song et al. 2010). Stakeholders discussed the potential tradeoffs among the human activities and the derived ES. For example, pollutant sources, such as nutrients could positively impact the primary production that influences the recreational fishery through food web and habitat alterations. However, algal production has negative impacts on beach aesthetics and may harbor waterborne pathogens (Whitman et al. 2003) (Table 1).

The last pathway (Table 1) illustrates how shipping and transporting goods and the associated HWB heavily relies on the ecosystem for its physical structure, navigational routes, and freshwater (lake levels) (Rothlisberger et al. 2012). Shipping contributes significantly to the local economy by providing jobs and transporting goods (e.g., coal) (Martin Associates 2011); however, shipping activities can introduce aquatic invasive species. Ships release ballast water for stabilization purposes, which may contain non-native mussels, algae, fish, and pathogens (Mills et al. 1993). The best known example is the invasion of zebra (*Dreissena polymorpha*) and quagga (*D. rostriformis bugenis*) mussels in the mid 1980s and 1990s, which has been significantly impacting the lake’s ecological structure and function (Nalepa and Gauvin 1988; Nalepa et al. 1996; David et al. 2009). Federal ballast water regulations have since been put in place to prevent introduction of aquatic invasive species.

### Phase III: Coupling Water Use, Tourism, and Ecological Indicators

**Fig. 3:**
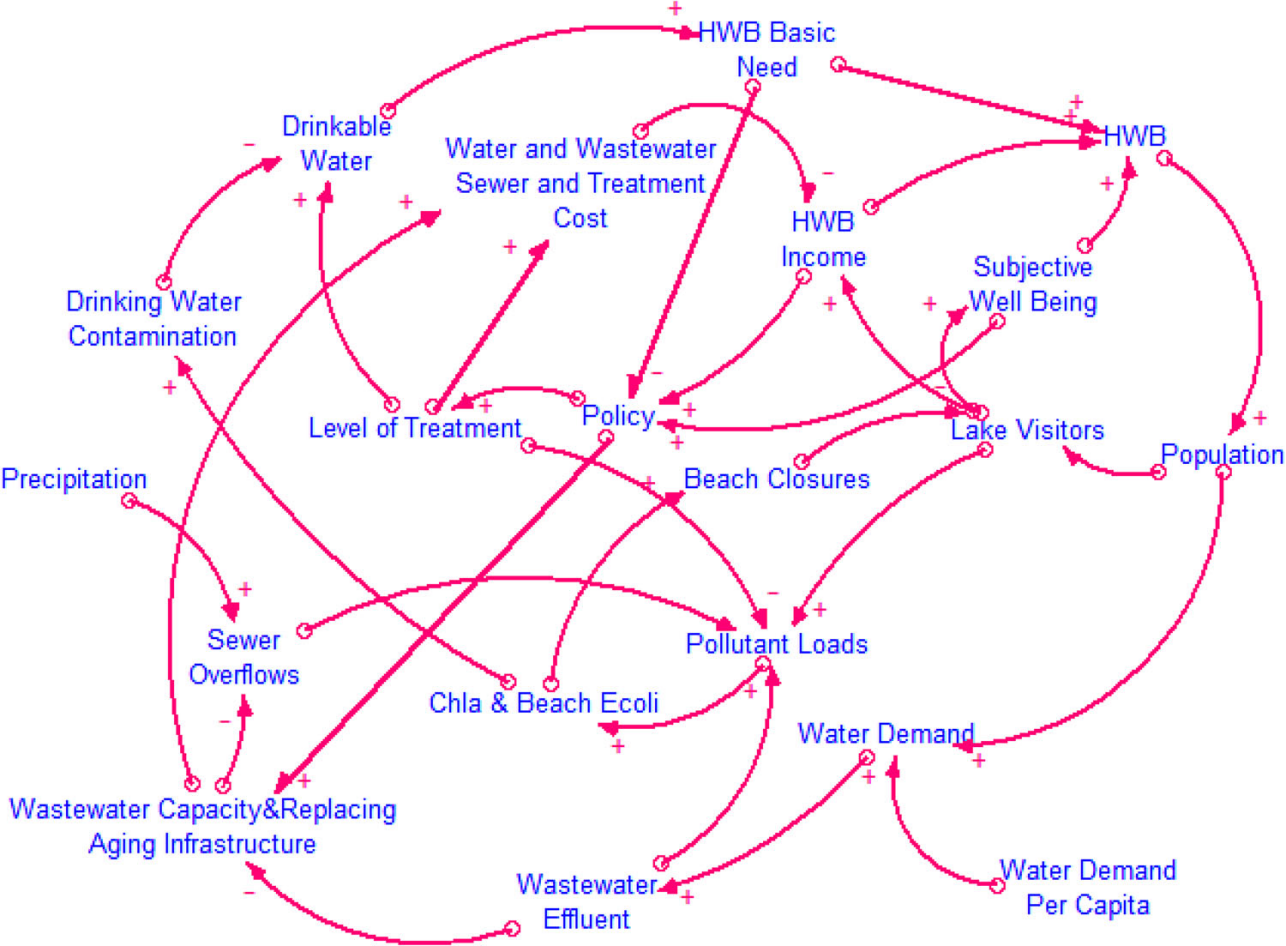
A causal loop diagram that represents Pathways 1 and 3 (see Table 1), which are residential water demand and tourism, and their impacts on the lake’s ecological condition

A snapshot of the interactions among the systems’ components was developed to show the complexity in developing integrative models (Fig. 2; Table 1). To illustrate how our conceptual framework can be transformed into a system dynamics model, we describe in detail the first pathway (human residential water use) and how it relates to the third pathway (tourism) by developing a CLD (Fig. 3). The complexities and feedbacks between the systems in CLD help reinforce the notion that conceptual frameworks are an essential prerequisite when building system dynamics models that can help operationalize sustainability.

HWB is defined through four components based on a recently proposed classification scheme that captures physical, mental, and social aspects of well-being (Summers et al. 2012): basic human needs, economic needs, environmental needs, and subjective well-being. In our simplified example, the two ES, freshwater—drinking water (basic human need) and recreation (subjective well-being and income), contribute to HWB.

**Fig. 4:**
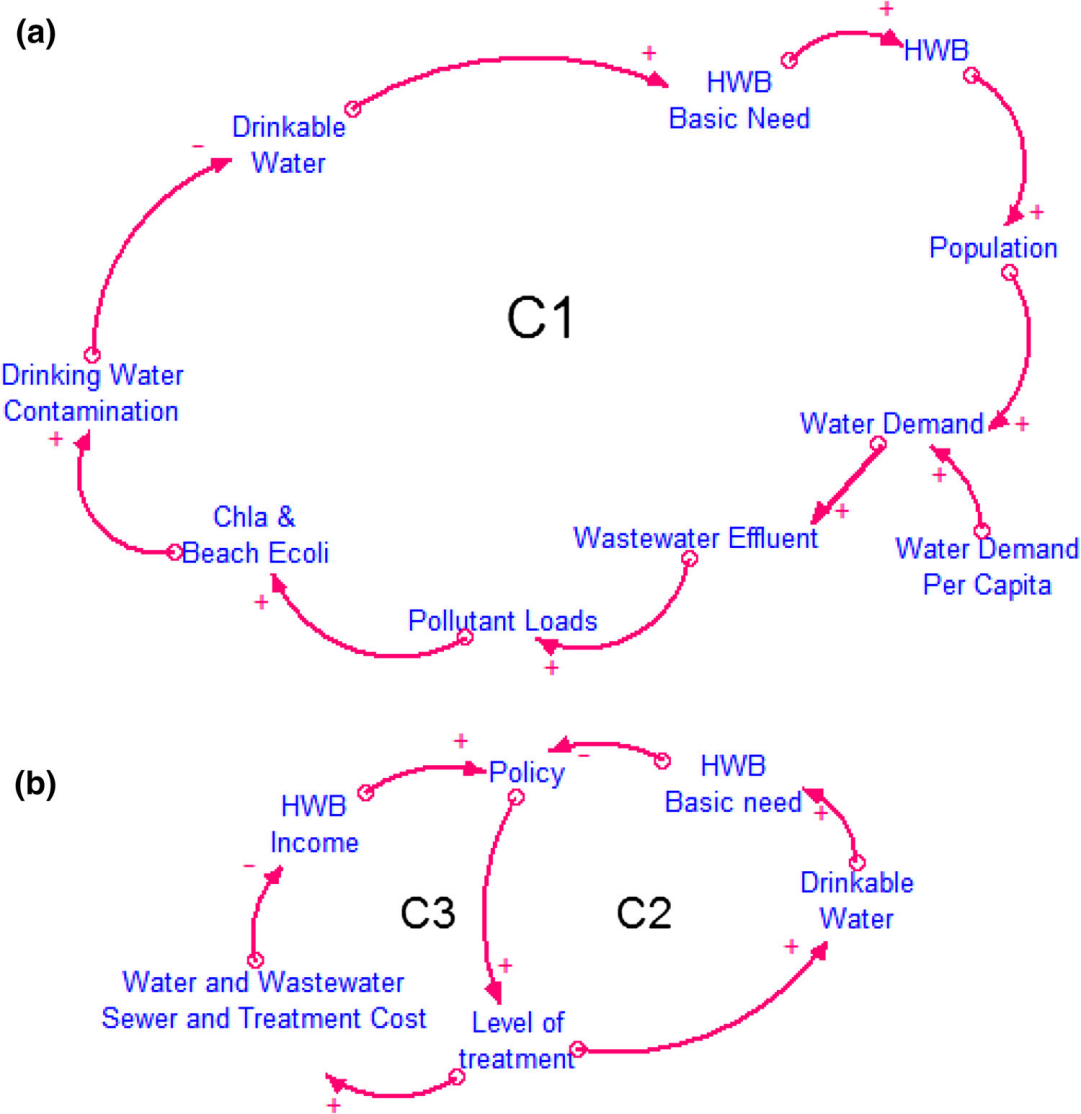

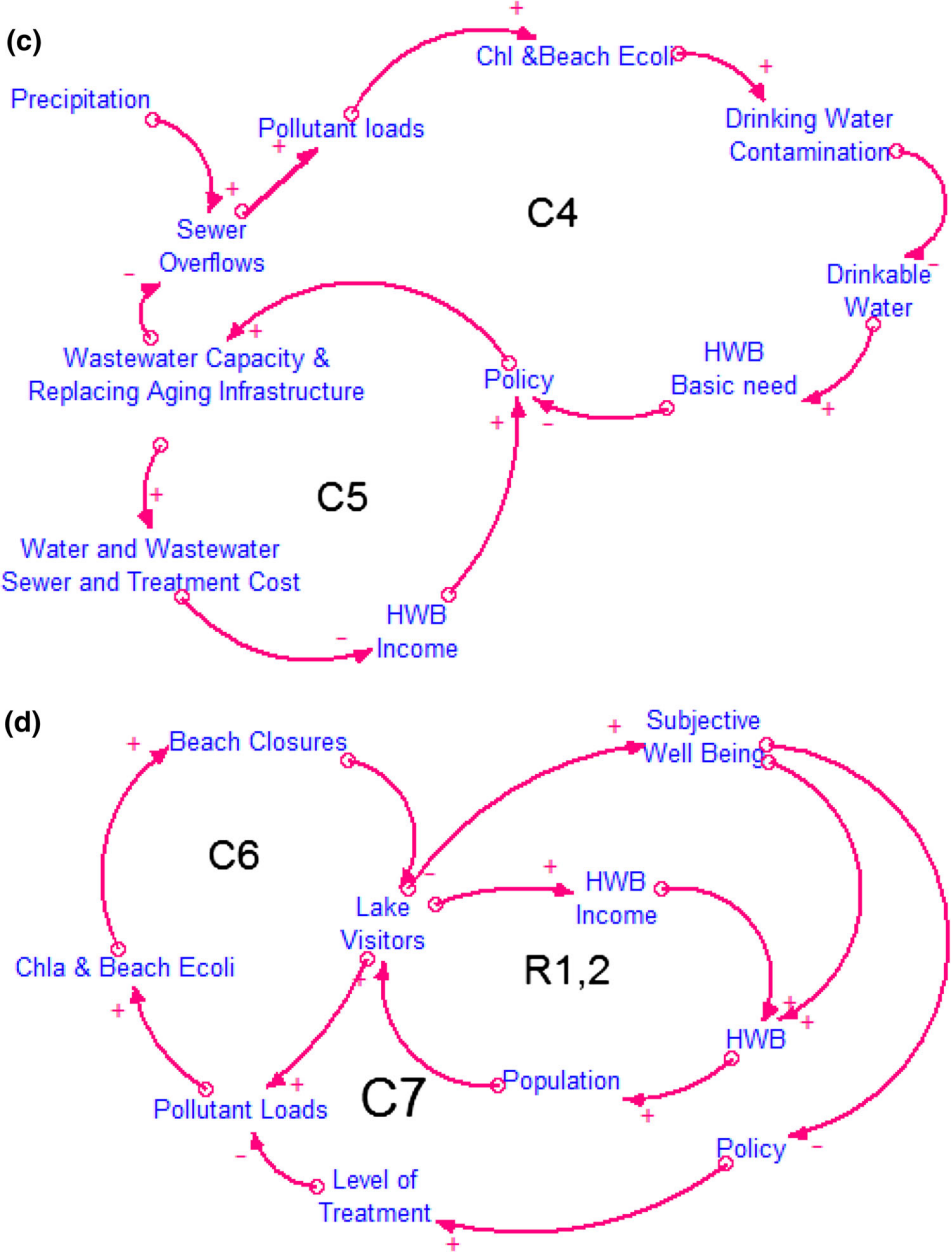
Details of the loop diagrams that represent Pathways 1 and 3 (see Table 1), the human activities of residential water demand and tourism. The panels include: **a** C1; **b** C2, C3; **c** C4, C5; **d** C6, C7, R1,2. Reinforcing or positive feedback loops, which are symbolized with “R”, represent a feedback loop that reinforces the original change. Counteracting or negative feedback loops, “C”, counteract the original change (Sterman 2000)

Figure 4 shows nine loops controlling the interplay between human activities and the ecological condition of freshwater ecosystems. The positive (+) arrows represent a cause and effect relationship in which the two parameters change in the same direction, while the negative (−) arrows represent two parameters that change in the opposite direction (Ford 1999).

#### Explanation of the Feedback Loops

Human population drives the water use that produces point source pollutant loads in the Clinton River watershed (Fig. 4a). Indicative determinants of the volume of wastewater and the pollutant loads entering the Clinton River and LSC include: residential water consumption per capita or household, number of served people or households, water use per type of industrial product and other activities, and available treatment technology. For simplicity reasons, the CLD only contains residential water demand per capita as an exogenous parameter. Discharged pollutants (e.g., nutrients and waterborne pathogens) enter the lake through the Clinton River and the spillway (Table 1). The impact from these pollutants can be measured by ecological responses of primary production (macrophyte distribution and phytoplankton biomass indicated by chlorophyll *a*) and concentrations of the fecal indicator bacteria *Escherichia coli* (*E. coli*). High concentrations of chlorophyll *a* and *E. coli* impact the flow of ES such as drinking water or recreation, which in turn both affect HWB that drives the population size (Fig. 4a, C1). At the same time, if the ES of drinking water is not addressed under the current conditions, policymaking is activated to protect public health. In this case, two opposing dimensions of HWB affect the policymaking process, the satisfaction of basic human needs such as drinking water and economic needs (utility) such as income. Policymaking can set the legislative framework for employing the appropriate water and/or wastewater treatment infrastructure to mitigate the effects of human activities on water quality and protect human health (Fig. 4b, C2). In this case, the most relevant policy action is revision of National Pollutant Discharge Elimination System permits to require greater treatment of effluent. If state water quality standards are violated, development of a Total Maximum Daily Load (TMDL) is required; the TMDL allocates pollution loads among point and nonpoint sources with the goal of achieving overall pollutant load reductions. However, the process of policymaking that might lead to the adoption of new technologies (higher levels of water and wastewater treatment) or replacing aging infrastructure is subject to the induced cost for the residents. More advanced methods of treatment lead to higher water and wastewater treatment costs (negative arrow), and the human population is left with lower income (and well-being), which would encourage a policy response to ease costs (Fig. 4b, C3).

Sewer overflows arising from CSOs along with aging infrastructure are considered by stakeholders to be the most serious sources of pollution in the study area (Fig. 4c, C4). Sewer overflows can increase pollutant loads which in turn can decrease the lakes’ ecological condition (increased concentrations of chlorophyll *a* and beach E. *coli*) resulting in drinking water contamination and beach closures due to violations of beach quality regulations. Drinking water contamination reduces the availability of drinkable water, which is a major human need, and results in the decrease of HWB. When basic human needs such as drinking water cannot be satisfied, policymaking for controlling sewer overflows is activated (Fig. 4c, C4). However, the control of sewer overflows increases the sewer cost implying that residents are left with a lower income (HWB income), which also triggers policymaking (Fig. 4c, C5).

Irrespective of the source of pollutant loads, violation of beach quality regulations leads to beach closures and decreases lake visitors and in turn the pollutants generated from direct beach usage (Fig. 4d, C6). At the same time, two reinforcing feedback loops related to subjective well-being and income are positively connected to beach usage (lake visitors), and increase the overall HWB, population in the area and the number of lake visitors (Fig. 4d, R1,2). Lastly, as lake visitors decrease and subjective well-being decays, new policy measures are needed to increase the level of treatment, to decrease pollutant loads that impact ecological condition (chlorophyll *a* and beach *E. coli)*, and to decrease the beach closures for increasing lake visitors (Fig. 4d, C7).

The parameters used in our example represent specific aspects of socio-ecological systems. Defining numerical values for those parameters is a challenge as some of them are uncertain. However, sensitivity and policy analysis can help determine if the estimated values for uncertain parameters reproduce the same pattern of results and if a policy creates the desired outcome (Ford 1999). This example represents the policy response only through the water and wastewater infrastructure (e.g., treatment technology or replacing aging infrastructure) ensuring safe drinking water and clean beaches for the local communities.

The cost for employing the appropriate infrastructure can be considered as an indirect payment for ES (e.g., drinking water). Other policy instruments might be capable of mitigating human effects on water quality such as government incentive payments, voluntary payments, and institutional changes (Brauman et al. 2007; Daily et al. 2009; Molnar and Kubiszewski 2012). The ability of those instruments to foster the maintenance of ES in the long run needs further investigation.

## Discussion

Our research reveals that the four proposed pathways are highly dependent on each other, implying that deriving the desired ES from the natural environment requires holistic, integrated management of the pathways in the systems. For example, developing and sustaining tourist activities (Pathway 3) will depend on management of point and non-point sources of pollution (Pathways 1 and 2). Another example is the impact of shipping through the discharge of ballast water which can introduce non-indigenous species (Pathway 4) that impact local ecological condition and subsequently can impact tourism and increase costs of maintaining water infrastructure. In our case study, water quality issues such as *E. coli* and algal blooms, create constraints on the tourist activities that directly depend on ecosystem condition. Conversely, there are human activities such as water use, boating and fishing that disturb the natural environment. Evaluating the potential tradeoffs between the benefits and costs among various human activities with respect to ecological condition and services can enable decision makers to manage valuable aquatic resources (Kremen and Ostfeld 2005). However, when evaluating the tradeoffs among ES, an important criterion is to include uncertainty for two main reasons. First, it remains a challenge to assess the full value of ecosystems in providing services (Brauman et al. 2007). Second, even if we can estimate the value that current users place in ES, it not possible to do so for future generations, implying the need to maintain the full range of services provided by the ecosystems (Bithas 2011).

Building CHANS frameworks for specific areas and defining the key parameters and data needs by eliciting stakeholder knowledge enhances our ability to develop dynamic models that capture real world systems (Carpenter et al. 2009). From the stakeholder workshop, we found our proposed framework can be used as a basis for further discussion and collaboration among interested parties as it ultimately suggests a holistic approach for watershed management and sustainability. Stakeholders’ knowledge helped our team to identify more pathways (e.g., Pathway 4), define essential feedbacks among systems’ components (e.g., arrows in Fig. 4) and explore potential policy management options. In terms of system dynamics, stakeholders provided us with the “casual knowledge” on how systems function and are interrelated (Jones et al. 2011).

We developed a CLD to present the complexity and the various feedback loops underlying water use and some aspects of recreation (beach usage defined through lake visitors). As long as the human population increases, the socioeconomic activities to serve this population will increase and as a result there will be increasing pressures on the natural systems. Maintaining aggregate HWB requires policy actions. In our example, the determinant parameter to mitigate the effects of human activities on water quality and maintain the ES of drinking water and recreation is the establishment of relevant pollution-prevention policies and construction and maintenance of appropriate infrastructure. Investment in infrastructure is critical for addressing sewer overflows, inadequate treatment, leaking pipes, and stormwater discharges, which are the major stressors in the study area. SEMCOG (2001) estimates that southeast Michigan will need to invest $14–26 × 10^9^ to maintain and improve the current sewer system and to remediate overflows. Although water and WWTPs can provide a substitute for some ES (i.e., waste processing) that maintain water quality for human consumption and recreation, pollution prevention strategies are still required to improve other measures of water quality to protect other ES. The quality of source water entering treatment plants also affects treatment and the drinking water quality that local communities enjoy (Levy et al. 2012). Although residents do not directly pay for ES, they pay indirectly for their loss in terms of substitutes (Summers et al. 2012) such as treatment technologies and the operation of water and WWTPs. The costs of substitutes may be large and some ES have no substitutes or technological fixes. This implies that people who mostly suffer the impacts of ecosystems’ deterioration are those who cannot employ instruments like technology to mitigate water quality issues. In some cases, technologies may have “lower resilience, cost effectiveness, suitability and life span than the ES they replace” (Brauman et al. 2007, p. 15). For example, the cities of New York and Boston found that watershed protection was more beneficial than constructing and maintaining filtration plants. Multiple groups in Michigan have been promoting green infrastructure as a cost-effective means of reducing pollutant loads.

Although we can conceptually and qualitatively define the key components of HWB, it remains a challenge to quantify the impacts of ES on HWB. The inability to quantitatively link ES and HWB can be viewed as a limitation as it constrains what can be represented in typical models. Approaches like creating CLDs by using system dynamics methodology allows to focus on critical linkages within CHANS. This can be useful for policy and for targeting research to get the necessary data for HWB quantification. These complex systems or case studies and data limitations should not prevent us from thinking about and fostering solutions to achieve sustainability. Our next step is to develop a system dynamics model based on our conceptual framework to move beyond conceptual linkages between socioeconomic and natural systems and to evaluate causal relationships (Sterman 2012). This dynamic model can provide decision makers useful tools for attaining sustainability under alternative scenarios.

## Conclusions

Designing for and achieving sustainability demands interdisciplinary approaches (Kremen and Ostfeld 2005) that fully integrate the knowledge of socioeconomic and natural sciences (Mavrommati and Richardson 2012). We suggest that evaluating the systems as a whole can enhance understanding of the importance for maintaining the functions and processes of natural systems for the healthy function of the socioeconomic system. Applying the concept of sustainability at an operational level remains a challenge given that increasing the current generations’ HWB arises from the degradation of ES (Raudsepp-Hearne et al. 2010). Maintaining certain components of the natural system by managing the socioeconomic systems’ activities is necessary for sustaining key ES that contribute to the well-being of current and future generations.

## Electronic supplementary material

Below is the link to the electronic supplementary material.

Supplementary material (PDF 235 kb)
