# Supplementary material for: Coupling Socioeconomic and Lake Systems for Sustainability: A Conceptual Analysis Using Lake St. Clair Region as a Case Study
Source: Ambio. 2013 Aug 15;43(3):275–87. doi: 10.1007/s13280-013-0432-4 (PMC3946116; doi:10.1007/s13280-013-0432-4)
Supplement: Supplementary file 1 — Supplementary material (PDF 235 kb) [file 13280_2013_432_MOESM1_ESM.pdf]

Electronic Supplementary Material

Coupling Socioeconomic and Lake Systems for Sustainability: A Conceptual Analysis Using Lake St. Clair Region as a Case Study

Georgia Mavrommati, Melissa M. Baustian and Erin A. Dreelin

Appendix S1: Exercise given to workshop participants

In our study we need your expertise to better understand the key variables and interactions taking place between human and lake systems (western basin of Lake St. Clair near the mouth of the Clinton River) in order to refine our conceptual model.

Lake St. Clair Workshop Exercise:

On the next page is a conceptual model that represents the framework for the workshop.

Please print this model, **complete it** and **bring it** with you to the workshop because we will be using it during the 3 workshop exercises (blank copies will be also available).

Note: we assume you will not know all parts of the conceptual model.

Helpful Hint: ask yourself three basic questions:

1. What are the basic ecosystem health indicators of the western basin of Lake St. Clair?
2. Which socioeconomic activities in the Clinton River watershed affect those ecosystem health indicators?
3. How does the ecological condition of the western basin of Lake St. Clair affect human well-being?

Conceptual Model Instructions:

1. Please **list the parameters** in the boxes of the socioeconomic and lake systems.
2. **Add arrows** to show how the boxes affect each other.

Definitions of Key Terms:

Ecosystem services are defined as the benefits people derive, directly or indirectly from the ecosystem functions (e.g. drinking water, fishing, storm protection).

Human well-being is defined as the quality of life people experience.

Lake St. Clair Workshop Exercise – May 18, 2012

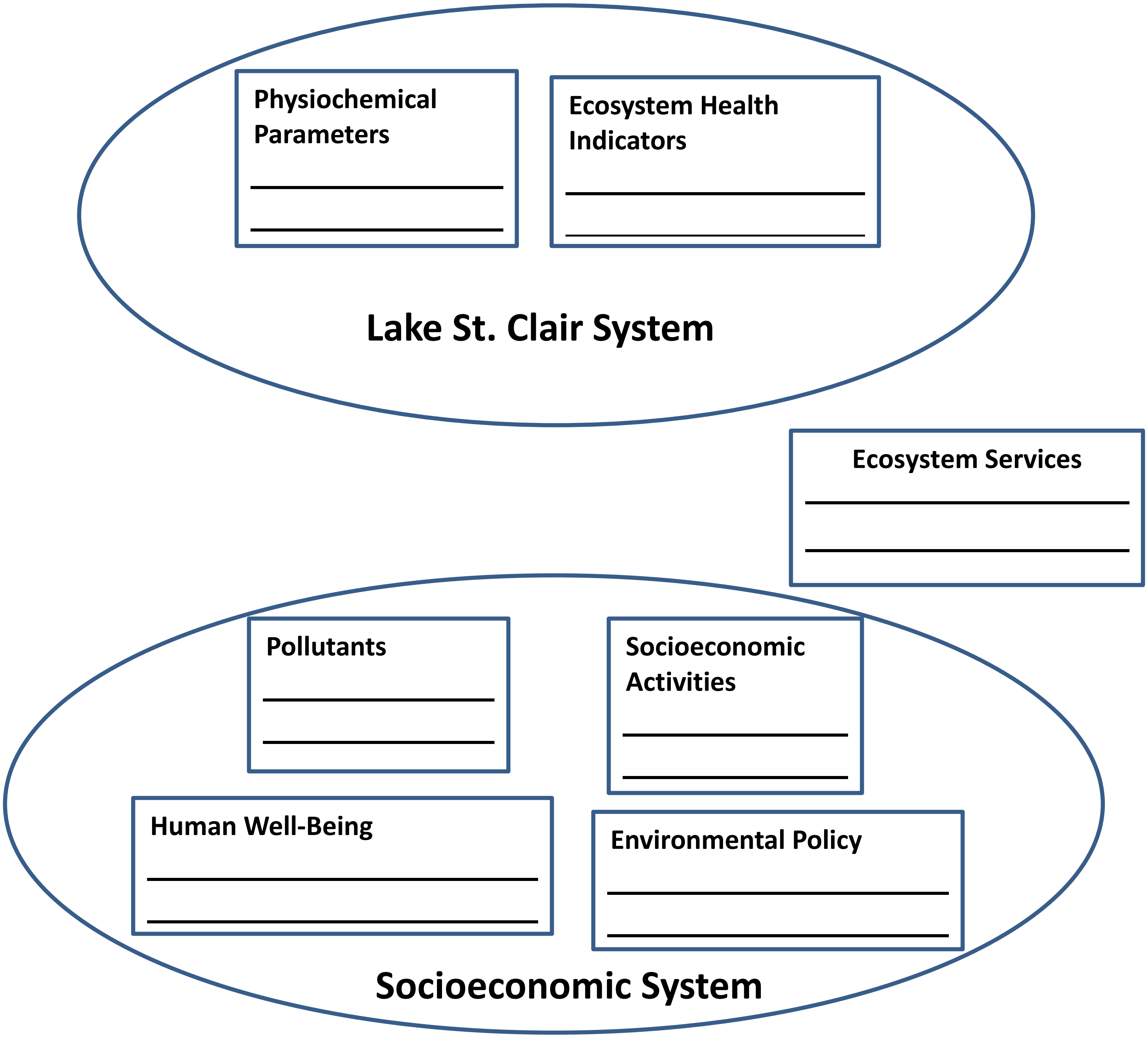

Conceptual Model Instructions:

1. Please **list parameters** in the boxes of the socioeconomic and lake systems.
2. **Add arrows** to show how the boxes affect each other.
